# Thermo-Responsive Behavior of Mixed Aqueous Solution of Hydrophilic Polymer with Pendant Phosphorylcholine Group and Poly(Acrylic Acid)

**DOI:** 10.3390/polym13010148

**Published:** 2021-01-01

**Authors:** Hirokazu Fukumoto, Kazuhiko Ishihara, Shin-Ichi Yusa

**Affiliations:** 1 Department of Applied Chemistry, Graduate School of Engineering, University of Hyogo, Hyogo 671-2280, Japan; climb.up.fk1685@gmail.com; 2 Department of Materials Engineering, School of Engineering, The University of Tokyo, Tokyo 113-8656, Japan; ishihara@mpc.t.u-tokyo.ac.jp

**Keywords:** hydrogen bond, thermo-responsive polymer, UCST, RAFT, salting out

## Abstract

A mixed aqueous solution of hydrophilic poly(2-methacryloyloxyethyl phosphorylcholine) (PMPC) and poly(acrylic acid) (PAAc) becomes cloudy under acidic conditions at room temperature. The pendant carboxylic acid groups in PAAc form hydrogen bonds with the ester and phosphate groups in PMPC. While the polymers aggregate under acidic conditions, neither one associate under basic conditions because of the deprotonation of the pendant carboxy groups in PAAc. We observed that the interpolymer complex formed from PMPC, and PAAc was dissociated in aqueous solutions with increasing temperature, which is an upper critical solution temperature behavior. With increasing temperature, the molecular motion increased to dissociate the interpolymer complex. The phase transition temperature increased with increasing polymer and salt concentrations, and with decreasing pH.

## 1. Introduction

**Figure 1:**
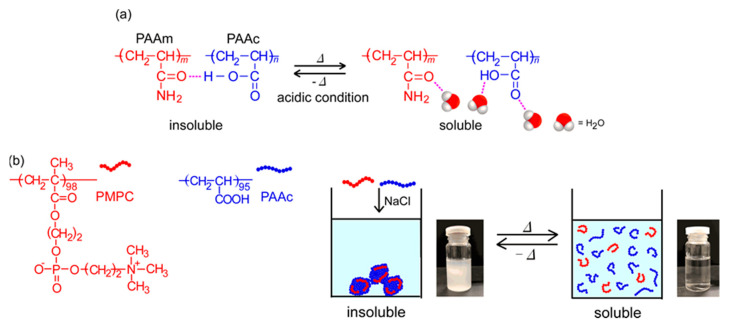
(**a**) Complex formation of poly(acrylamide) (PAAm) and poly(acrylic acid) (PAAc) owing to hydrogen bonding interactions, and the dissociation of aggregates upon heating; and (**b**) conceptual illustration of the UCST behavior of the mixed aqueous solutions of poly(2-methacryloyloxyethyl phosphorylcholine) (PMPC) and PAAc.

Stimuli-responsive polymers can change their physical and/or chemical properties on exposure to external conditions, such as temperature, pH, salt concentration, light, and magnetic fields [1,2,3,4]. A thermo-responsive polymer, poly(*N*-isopropylacrylamide) (PNIPAM), has been found to become insoluble in water owing to dehydration above the lower critical solution temperature (LCST) [5]. PNIPAM is used widely in the biomedical and bioengineering fields because the phase transition temperature (*T*_p_; ~32 °C) is close to the human body temperature at physiological concentrations [6,7,8]. Generally, the *T*_p_ of PNIPAM increases and decreases to copolymerize hydrophilic and hydrophobic monomers, respectively [9]. It is well-known that another class of thermo-responsive polymers with the upper critical solution temperature (UCST) shows phase separation below the UCST. Above the UCST, the polymer is soluble in the solvent. However, thus far, only a few UCST water-soluble polymers have been reported, compared to LCST polymers [10]. UCST-type phase separation behavior in aqueous media can be observed by electrostatic [11] and/or hydrogen bonding interactions [12]. Zwitterionic sulfobetaine polymers are insoluble in water below the UCST because of the formation of aggregates by electrostatic attractive interactions. Conversely, above the UCST, the sulfobetaine polymers are soluble because the molecular motion of the polymer chains overcomes the charge interactions. Random copolymers composed of acrylamide and acrylonitrile become insoluble in water at low temperatures because of the hydrogen bonding interactions between the polymer chains, and they become soluble with increasing temperature because of the breakdown of hydrogen bonds [13,14]. The above examples of UCST polymers are single-component homopolymers or random copolymers in aqueous solutions. It is known that a mixture of two water-soluble polymers shows the UCST [15]. When poly(acrylamide) (PAAm) and poly(acrylic acid) (PAAc) homopolymers are mixed in water under acidic conditions at room temperature, the pendant carbonyl group in PAAm forms a hydrogen bond with the pendant carboxylic acid in PAAc and precipitates (Figure 1a). The molecular motion increases, and the polymer aggregates are dissociated with increasing temperature because the hydrogen bonds between PAAm and PAAc break [16,17,18]. The proton in the carboxylic acid in PAAc acts as a hydrogen donor. Therefore, the UCST behavior of PAAm/PAAc can be observed only in acidic conditions. PAAc shows pH-responsive behavior owing to hydration and dehydration of the pendant carboxy groups [19,20,21]. Poly(2-methacryloyloxyethyl phosphorylcholine) (PMPC) is a biomimetic material possessing a pendant hydrophilic phosphorylcholine group, which has the same structure as the surface of cell membranes. PMPC can be applied to biological and medical technology in the field of nanomaterials. PMPC is applied in medical fields for artificial joints, catheters, contact lenses, etc., [22,23,24].

In this study, by reversible addition-fragmentation chain transfer (RAFT) radical polymerization, we prepared PMPC and PAAc with degrees of polymerization (DP) of 98 and 95, respectively. At pH 3, the pendant carboxy groups were protonated. To monitor the interactions between PMPC and PAAc, the values of percent transmittance (%*T*) were measured for the mixed aqueous solutions of PMPC and PAAc with varying molar ratios (*f*_AAc_ = [AAc]/([MPC] + [AAc])), where [AAc] and [MPC] are the molar concentrations of the acrylic acid (AAc) and 2-methacryloyloxyethyl phosphorylcholine (MPC) units in the aqueous solution, respectively. The PMPC/PAAc complex was formed at pH 3 because the pendant carbonyl and phosphate groups in PMPC formed hydrogen bonds with the pendant carboxylic acid in PAAc. At *f*_AAc_ = 0.85, PMPC and PAAc formed a complex and precipitated. We studied the UCST behavior of PMPC/PAAc complex aqueous solutions (Figure 1b). The *T*_p_ of the PMPC/PAAc complex aqueous solutions increased with increasing NaCl concentration ([NaCl]) and polymer concentration (*C*_p_), and *T*_p_ decreased with increasing pH.

## 2. Materials and Methods

MPC (NOF Co., Tokyo, Japan) was recrystallized from acetonitrile. AAc (98%) from FUJIFILM Wako Pure Chemical Co. (Osaka, Japan) was dried with a 4A molecular sieve (Kanto Chemical Co. Tokyo, Japan) and distilled under reduced pressure. 4,4′-Azobis-(4-cyanovaleric acid) (V-501; 98%) and 2,2′-azobisisobutyronitrile (AIBN; Wako, 98%) from FUJIFILM Wako Pure Chemical Co. (Osaka, Japan) were recrystallized from methanol. 4-Cyanopentanoic acid dithiobenzoate (CPD) was synthesized according to a procedure reported previously [25]. Methanol (99.9%) was dried with a 4A molecular sieve (Kanto Chemical Co. Tokyo, Japan) and distilled before use. Water with an ion-exchange column was used throughout the study.

### 2.1. Preparation of PMPC

MPC (5.08 g, 17.2 mmol), CPD (47.5 mg, 0.170 mmol), and V-501 (19.1 mg, 0.0681 mmol) (mole ratio 100:1:0.4) were dissolved in a water/methanol solvent (17.0 mL, 9/1, *v*/*v*). The solution was purged with argon gas, stirring for 30 min to remove the oxygen. After that, the solution was heated at 70 °C for 4 h. The monomer conversion was 98.5%, as estimated from proton nuclear magnetic resonance (^1^H NMR, Bruker, Billerica, MA, USA) before purification (Figure S1). The reaction mixture was dialyzed against pure water for one day. The polymer (PMPC) was recovered by a freeze-drying technique (4.34 g, 85.4%). The values of the number-average molecular weight (*M*_n_(GPC)) and the molecular weight distribution (*M*_w_/*M*_n_), as estimated from gel-permeation chromatography (GPC, Tosoh Co. Tokyo, Japan) measurements were 27.9 kDa and 1.37, respectively. The degree of polymerization calculated from ^1^H NMR (DP(NMR)) was 98.

### 2.2. Preparation of PAAc

AAc (5.86 g, 81.4 mmol), CPD (87.5 mg, 0.343 mmol), and AIBN (22.7 mg, 0.138 mmol) (mole ratio 237:1:0.4) were dissolved in methanol (80.0 mL). The solution was purged with argon gas, stirring for 30 min to remove the oxygen. After that, the solution was heated at 60 °C for 44 h. The monomer conversion was 39.8%, estimated from ^1^H NMR before purification (Figure S2). After polymerization, the solution was dialyzed against pure water for one week. The polymer (PAAc) was recovered by a freeze-drying technique (0.936 g, 16.2%). The *M*_n_(GPC) and *M*_w_/*M*_n_ were 9.10 kDa and 1.42, respectively. The theoretical degree of polymerization (DP(theo)) calculated from the conversion was 95.

### 2.3. Measurements

^1^H NMR spectra were obtained using a Bruker (Billerica, MA, USA) DRX-500 spectrometer. GPC measurements were performed using a Tosoh (Tokyo, Japan) RI-8020 reflective index detector, Tosoh DP-8020 pump, and Shodex (Tokyo, Japan) GF-7M column. A phosphate buffer (50 mM) at pH 9 and acetonitrile mixed solvent (9/1, *v*/*v*) was used as the eluent at 40 °C. The *M*_n_(GPC) and *M*_w_/*M*_n_ values for the polymers were calibrated using standard sodium poly(styrene sulfonate) samples. pH titration was performed using a Hiranuma Sangyo (Osaka, Japan) COM-1600 auto-titrator equipped with a glass electrode in 4.0 M KCl. PAAc was dissolved in 0.1 M NaOH at *C*_p_ = 5.0 g/L, which was titrated using 0.1 M HCl_aq_. Ultraviolet–visible spectra were obtained using a Jasco (Tokyo, Japan) V-730 UV–vis spectrophotometer at 700 nm. To determine the UCST, the temperature was controlled using a Jasco ETC-717 temperature controller at a cooling rate of 1.0 °C/min. The *T*_p_ was defined as the temperature at which the %*T* at 700 nm starts to decrease.

## 3. Results and Discussion

### 3.1. Preparation and Characterization of the Polymers

To prepare PMPC and PAAc with similar DP values, we used a RAFT technique using a dithiobenzoate chain transfer agent (CTA) for controlled radical polymerization. The conversions (*p*) of PMPC and PAAc were 98.5% and 39.8%, respectively, estimated from ^1^H NMR after polymerization. The DP(theo) and theoretical *M*_n_ (*M*_n_(theo)) were calculated using the following formulas:

(1) $$\mathrm{DP}\left(\mathrm{theo}\right)=\;\frac{{\left[M\right]}_{0}}{{\left[\mathrm{CTA}\right]}_{0}}\times \frac{p}{100}$$

(2) $$M_{n}\left(\mathrm{theo}\right)=\mathrm{DP}\left(\mathrm{theo}\right)\times M_{m}+M_{\mathrm{CTA}}$$

where [M]_0_ and [CTA]_0_ are the initial monomer and CTA concentrations, and *M*_m_ and *M*_CTA_ are the molecular weights of the monomer and CTA, respectively. The DP(theo) values for PMPC and PAAc were 99 and 95, respectively. ^1^H NMR spectra for PMPC and PAAc were measured in D_2_O at 20 °C. (Figure S3). The DP(NMR) for PMPC was 98, which was calculated from the integral intensity ratio of the pendant methylene protons at 3.7 ppm and the terminal phenyl protons attributed to CTA at 7.4–8.2 ppm. The DP(NMR) = 98 was close to the theoretical value, DP(theo) = 99. The DP(NMR) of PAAc was 104, as estimated from the integral intensity ratio of the main chain protons at 1.4–2.6 ppm and the terminal phenyl protons at 7.4–8.2 ppm, which was close to the DP(theo) = 95. GPC for PMPC and PAAc were measured using phosphate buffer as the eluent (Figure S4). The GPC elution curves for PMPC and PAAc were unimodal, and the *M*_w_/*M*_n_ values were 1.37 and 1.42, respectively, which indicated that the polymers had well-controlled structures. The DP, *M*_n_, and *M*_w_/*M*_n_ for PMPC and PAAc are summarized in Table 1.

PAAc was dissolved in 0.1 M NaOH (*C*_p_ = 5.0 g/L) and titrated against 0.1 M HCl to obtain the titration curve (Figure S5). The *x*- and *y*-axes show the volume of HCl added (Vol_HCl_) and the pH value of the solution, respectively. The end point (EP) indicates the neutralization point. The acid dissociation constant (p*K*_a_) value of PAAc was determined from the following equation [26]:

(3) $${\mathrm{Vol}}_{{\mathrm{EP}}_{1/2}}={\mathrm{Vol}}_{\mathrm{EP}}+\frac{1}{2}\frac{\left[\mathrm{COOH}\right]}{\left[\mathrm{HCl}\right]}{\mathrm{Vol}}_{\mathrm{sol}}$$

where Vol_EP_ is the volume difference of the HCl added at the two-step inflection point, [COOH] is the pendant carboxylic acid concentration in PAAc, [HCl] is the concentration of HCl, the titrant used, and Vol_sol_ is the initial volume of the polymer solution being titrated. In this measurement, the titration curve with two inflection points was obtained. As HCl was titrated, the first inflection point was due to the neutralization of NaOH, and the second was due to the protonation of PAAc. The HCl volumes used for the titration at the two inflection points were 1.2 and 3.7 mL, respectively, indicating that Vol_EP_ was 2.5 mL. The value of Vol_EP1/2_ was 3.3 mL, estimated from Equation (3). As the pH at Vol_EP1/2_ was pH_EP1/2_, this value corresponded to p*K*_a_. Therefore, from the titration curve, pH_EP1/2_ was 4.46 when Vol_EP1/2_ was 3.3 mL. The p*K*_a_ value of PAAc was determined to be 4.46. The estimated p*K*_a_ value of PAAc was close to the literature value (p*K*_a_ = 4.5) [27,28]. Hereafter, all experiments were performed at pH 3, where PAAc was protonated, unless otherwise noted. The protonation degree (δ) of PAAc in the aqueous solution was calculated based on the following equations [29,30]:

(4) $$\delta =\frac{1}{1+10^{\mathrm{pH}-pK_{a}}}$$

According to the equation, 96.6% of the pendant carboxy groups in PAAc were protonated.

### 3.2. Mole Ratio Dependence on Solubility after Mixing Polymers

**Figure 2:**
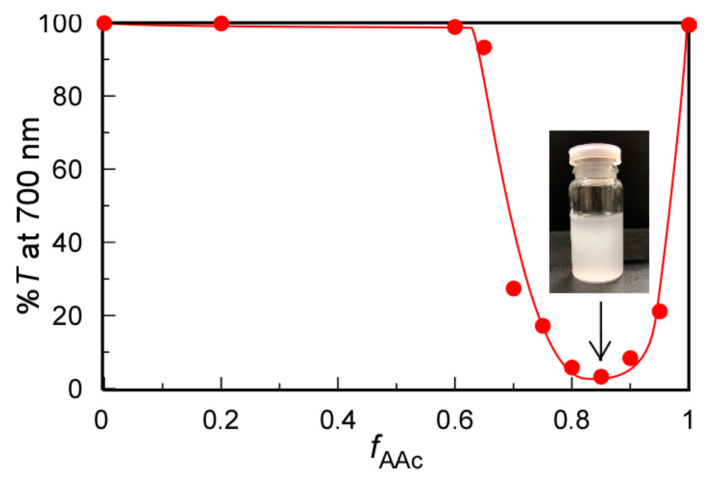
Percent transmittance (%*T*) at 700 nm for PMPC/PAAc mixed aqueous solutions as a function of *f*_AAc_ (= [AAc]/([MPC]+[AAc])) at *C*_p_ = 0.5 g/L, [NaCl] = 0.1 M, pH = 3, and 20 °C. The insert is a picture of the solution at *f*_AAc_ = 0.85.

At pH 3, the pendant carboxy groups in PAAc were protonated in 0.1 M NaCl aqueous solutions at 20 °C. We studied the solubility changes of the mixed aqueous solutions of PMPC and PAAc with varying *f*_AAc_ values using %*T* (Figure 2). At *f*_AAc_ ≤ 0.6, %*T* was constant at 100%. At *f*_AAc_ > 0.6, %*T* started to decrease, suggesting the formation of an insoluble PMPC/PAAc complex because the pendant carbonyl acceptor in the PMPC formed hydrogen bonds with the pendant carboxylic acid donor in PAAc. At *f*_AAc_ = 0.85, %*T* decreased to the minimum value of 4.1%, suggesting the formation of the largest PMPC/PAAc complex with the strongest interpolymer interactions. Furthermore, 5.7 pendant carboxylic acids in PAAc interacted with one pendant group in PMPC at *f*_AAc_ = 0.85, that is, [MPC]/[AAc] = 1/1.8. Surprisingly, interactions between PMPC and PAAc were not observed from %*T* at *f*_AAc_ = 0.5, that is, [MPC]/[AAc] = 1/1. This is because the pendant oxygen atoms in the ester and oxygen atoms in the phosphorylcholine group in PMPC can act as proton acceptors. Tupikina et al. reported that one P=O acceptor in phosphoric acid binds two hydrogen donors [31]. Hereafter, all experiments were performed at *f*_AAc_ = 0.85, unless otherwise noted.

### 3.3. pH Dependence on Solubility

**Figure 3:**
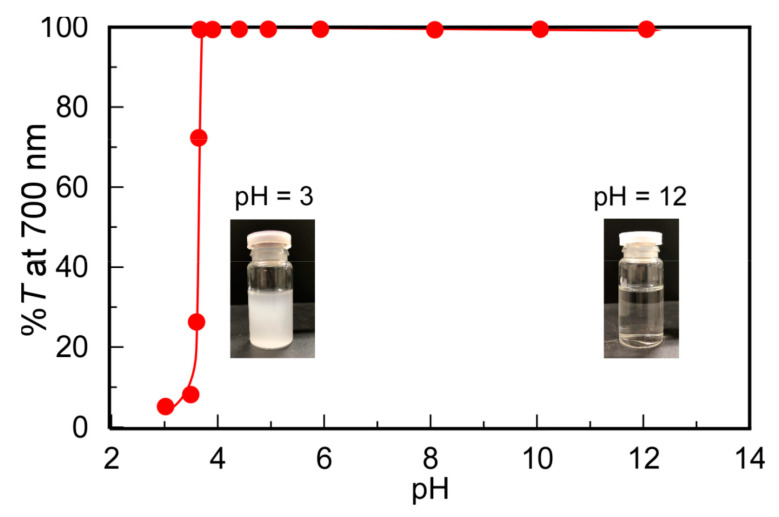
Percent transmittance (%*T*) at 700 nm for PMPC/PAAc mixed aqueous solutions with *f*_AAc_ = 0.85 as a function of pH at *C*_p_ = 0.5 g/L, [NaCl] = 0.1 M, and 20 °C. Inserts are pictures of the PMPC/PAAc aqueous solutions at pH 3 and 12.

The changes in solubility depending on pH were investigated for PMPC/PAAc with *f*_AAc_ = 0.85 (Figure 3). At pH 3, %*T* decreased to 5.2%, suggesting that the protonated pendant carboxylic acids in PAAc formed hydrogen bonds with the pendant ester and phosphorylcholine groups in PMPC to form insoluble PMPC/PAAc complexes. As the pH value increased from 3, the hydrogen bonding interactions of PMPC with PAAc were weakened because of the deprotonation of the pendant carboxylic acid groups in PAAc. Above pH 4, %*T* became 100%. The %*T* values of each aqueous solution of PMPC and PAAc were 100% from pH 3 to 12. These observations indicate that the complex formation of PMPC and PAAc originated from the hydrogen bonding interactions between the pendant groups in PMPC and PAAc below pH 4.

### 3.4. pH, Cp, and [NaCl] Dependence on UCST

**Figure 4:**
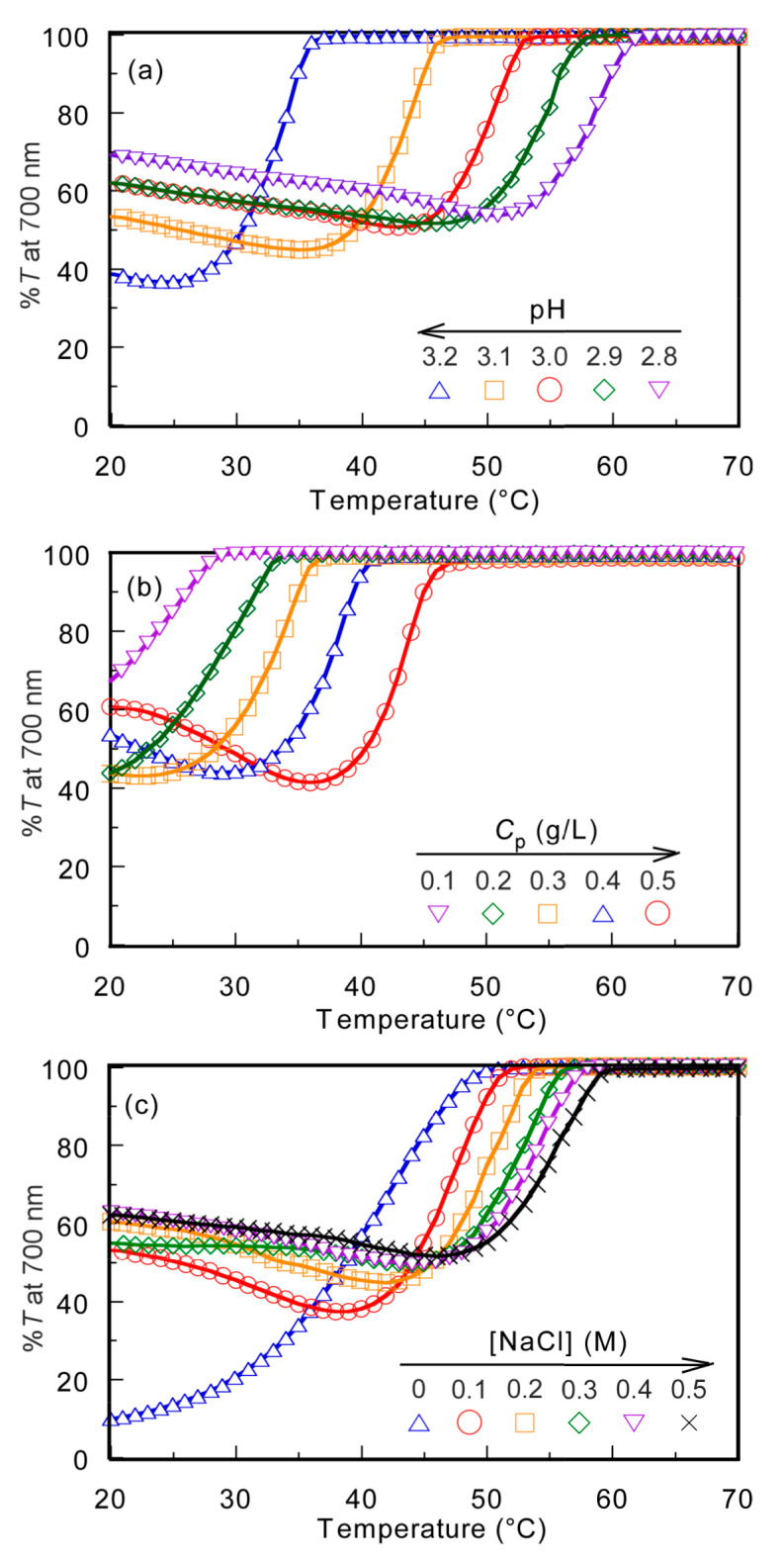
Percent transmittance (%*T*) at 700 nm for PMPC/PAAc mixed aqueous solutions with *f*_AAc_ = 0.85 as a function of temperature at pH 3, *C*_p_ = 0.5 g/L, and [NaCl] = 0.1 M, which is a standard condition: (**a**) pH, (**b**) *C*_p_, and (**c**) [NaCl] dependence on the phase transition behavior.

The PMPC/PAAc aqueous solution with *f*_AAc_ = 0.85 showed the UCST behavior at pH 3.0 and *C*_p_ = 0.5 g/L. The %*T* of the PMPC/PAAc aqueous solution with *f*_AAc_ = 0.85 was 100% at [NaCl] = 0.1 M, which started to decrease with the cooling process. Large hysteresis was observed for the plots of %*T* vs temperature with the heating and cooling processes (Figure S6). However, the plot of %*T* vs temperature with the cooling process always overlapped without hysteresis. Therefore, in this study, we focused on the cooling process of the UCST. The temperature at which %*T* decreased from 100% was defined as *T*_p_. We studied the effect of pH, *C*_p_, and [NaCl] on *T*_p_. The PMPC/PAAc aqueous solution with *f*_AAc_ = 0.85 at pH 3, *C*_p_ = 0.5 g/L, and [NaCl] = 0.1 M was used as the standard condition. *T*_p_ was determined from the change in %*T* as a function of temperature during the cooling process when one condition was changed and the other two were fixed (Figure 4).

**Figure 5:**
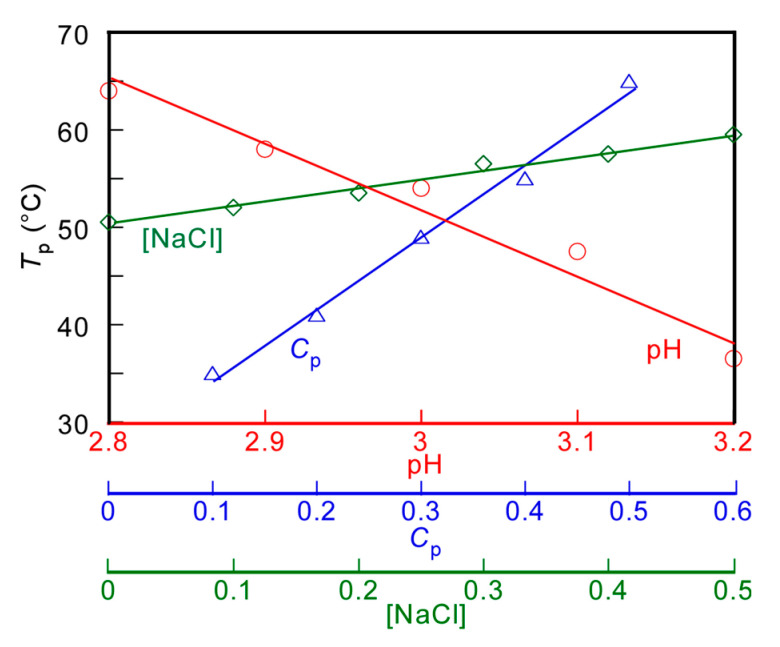
pH (○), polymer concentration (*C*_p_, △), and salt concentration ([NaCl], ◇) dependence on the phase transition temperature (*T*_p_) for aqueous PMPC/PAAc solutions with *f*_AAc_ = 0.85.

The *T*_p_ values of the PMPC/PAAc aqueous solution with *f*_AAc_ = 0.85 at *C*_p_ = 0.5 g/L and [NaCl] = 0.1 M were measured at varying pH values (Figure 4a). *T*_p_ became relatively low with increasing pH. With increasing pH, the deprotonation of PAAc hindered the formation of hydrogen bonds with PMPC, and low energy was enough to break the interpolymer interactions, leading to a decrease in *T*_p_. The *T*_p_ values were measured at varying *C*_p_ values (Figure 4b). The *T*_p_ value became relatively high with increasing *C*_p_. At high *C*_p_, there were numerous polymer chains, which resulted in a high probability of the formation of the PMPC/PAAc complex owing to hydrogen bonding interactions between polymer chains. Therefore, with increasing *C*_p_, more energy was required to break the hydrogen bonding interactions, which resulted in a significant increase in the *T*_p_ value. The trend where *T*_p_ increases with increasing *C*_p_ was observed for other UCST polymers, such as random copolymers of acrylamide and styrene in water [32]. The *T*_p_ values were measured in the range of 0–0.5 M [NaCl] (Figure 4c). The *T*_p_ value became relatively high with increasing [NaCl]. At low [NaCl], each polymer chain was hydrated with water molecules, increasing the polymer solubility in the aqueous solution. Conversely, at high [NaCl], dehydration of the polymer chains occurred; this was because NaCl took water molecules from the polymer chains. The dehydrated polymer chains easily formed relatively strong hydrogen bonds. With increasing [NaCl] content, more energy was required to break the hydrogen bonds. This resulted in a significant increase in the *T*_p_ value with increasing [NaCl]. The relationships of *T*_p_ with pH, *C*_p_, and [NaCl] are summarized in Figure 5.

### 3.5. Effect of Urea on the UCST

**Figure 6:**
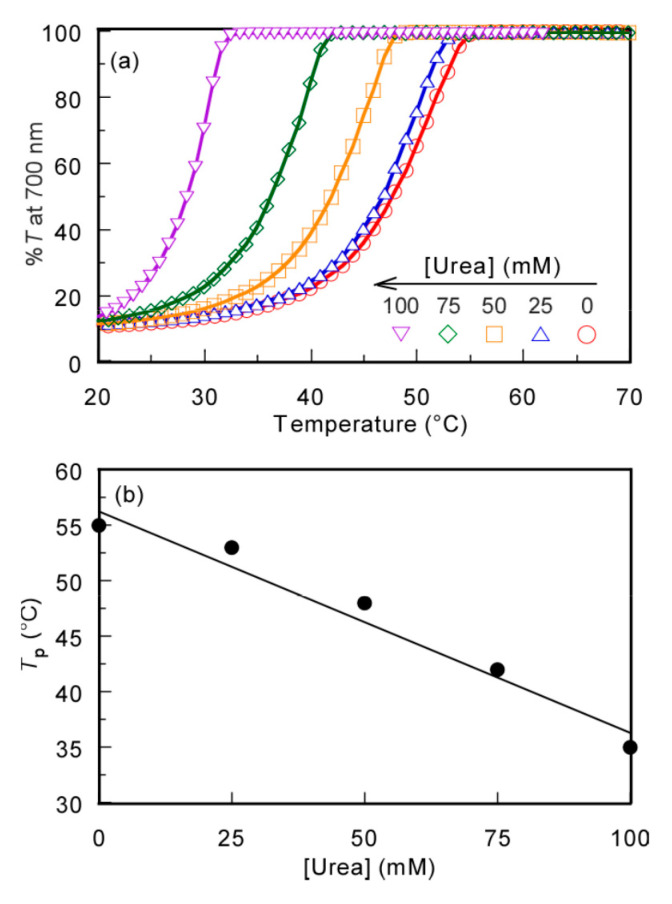
(**a**) Percent transmittance (%*T*) at 700 nm for the PMPC/PAAc mixed aqueous solution with *f*_AAc_ = 0.85 at *C*_p_ = 0.5 g/L, pH 3, and [NaCl] = 0.1 M as a function of temperature in the presence of urea; and (**b**) *T*_p_ as a function of urea concentration ([Urea]).

To confirm that the mechanism of the UCST behavior was due to the hydrogen bonding interactions, the effect of the hydrogen bond inhibitor, urea, on *T*_p_ was studied. The %*T* of the PMPC/PAAc aqueous solution with *f*_AAc_ = 0.85 at *C*_p_ = 0.5 g/L, [NaCl] = 0.1 M, and pH 3.0 was measured at varying urea concentrations ([Urea]) (Figure 6). The *T*_p_ values became relatively low with increasing [Urea]. The formation of hydrogen bonds between polymer chains was restricted by urea, and the polymer chains were hydrated to increase their solubility in aqueous solution [33]. This finding indicates that the hydrogen bonding interaction is the main driving force of the UCST behavior of the PMPC/PAAc complex in aqueous solutions.

## 4. Conclusions

PMPC and PAAc were prepared by RAFT radical polymerization. PMPC and PAAc formed complexes in aqueous solutions at pH 3 because of the hydrogen bonding interactions between the pendant ester and phosphorylcholine groups in PMPC and the pendant carboxylic acid group in PAAc. The strongest interactions between PMPC and PAAc can be observed at *f*_AAc_ = 0.85. The *T*_p_ value of the aqueous PMPC/PAAc solution increased with increasing *C*_p_ and [NaCl] and with decreasing pH. When urea, which is a hydrogen bond inhibitor, was added to the PMPC/PAAc aqueous solution, the *T*_p_ became relatively low. The main factor determining the behavior of the UCST is hydrogen bonding interactions.

## Figures and Tables

**Table 1:** Degree of polymerization (DP), number-average molecular weight (*M*_n_), and molecular weight distribution (*M*_w_/*M*_n_).

	DP(theo) ^a^	*M*_n_(theo) ^b^ kDa	DP(NMR) ^c^	*M*_n_(NMR) ^c^ kDa	*M*_n_(GPC) ^d^ kDa	*M*_w_/*M*_n_^d^
PMPC	99	29.5	98	29.2	27.9	1.37
PAAc	95	7.07	104	7.75	9.10	1.42

^a^ Calculated from Equation (1); ^b^ calculated from Equation (2); ^c^ estimated from ^1^H NMR; ^d^ estimated from GPC.

## Data Availability

Data is contained within the article or supplementary material.

## Supplementary Materials

The following are available online at https://www.mdpi.com/2073-4360/13/1/148/s1: Figure S1. ^1^H NMR spectra for MPC (a) before and (b) after polymerization in a water/methanol mixed solvent (17.0 mL, 9/1, *v*/*v*). The solution was added D_2_O to lock the NMR equipment. The NMR measurements were performed at room temperature; Figure S2. ^1^H NMR spectra for acrylic acid (a) before and (b) after polymerization in methanol. The solution was added D_2_O to lock the NMR equipment. The NMR measurements were performed at room temperature; Figure S3. ^1^H NMR spectra for (a) PMPC and (b) PAAc in D_2_O at 20 °C. Assignments are indicated for the resonance peaks; Figure S4. GPC elution curves for PMPC (―) and PAAc (---) using a mixed solvent of 50 mM phosphate buffer at pH 9 and acetonitrile (9/1, *v/v*) as an eluent at 40 °C; Figure S5. EP and EP_1/2_ positions on the PAAc titration curve at *C*_p_ = 5.0 g/L titrated against HCl in 0.1 M aqueous solution at 25 °C: Firstly, PAAc was dissolved in 0.1 M NaOH at *C*_p_ = 5.0 g/L; Figure S6. Percent transmittance (%*T*) at 700 nm for PMPC/PAAc with *f*_AA_ = 0.85 mixed aqueous solutions at pH 3, *C*_p_ = 0.5 g/L, and [NaCl] = 0.1 M as a function of temperature with the 2nd (circle) and 3rd (triangle) heating (red) and cooling processes (blue).
