# Supplementary material for: Thermo-Responsive Behavior of Mixed Aqueous Solution of Hydrophilic Polymer with Pendant Phosphorylcholine Group and Poly(Acrylic Acid)
Source: Polymers (Basel). 2021 Jan 1;13(1):148. doi: 10.3390/polym13010148 (PMC7794920; doi:10.3390/polym13010148)
Supplement: Supplementary file 1 [file polymers-13-00148-s001.pdf]

Supplementary Materials

Thermo-responsive Behavior of Mixed Aqueous Solution of Hydrophilic Polymer with Pendant Phosphorylcholine Group and Poly(Acrylic Acid)

Hirokazu Fukumoto[1], Kazuhiko Ishihara[2], Shin-ichi Yusa[1,]*

[1] Department of Applied Chemistry, Graduate School of Engineering, University of Hyogo, 2167 Shosha, Himeji, Hyogo, 671-2280, Japan; climb.up.fk1685@gmail.com

[2] Department of Materials Engineering, School of Engineering, The University of Tokyo, 7-3-1 Hongo, Bunkyo-ku Tokyo 113-8656, Japan; ishihara@mpc.t.u-tokyo.ac.jp

* Correspondence: yusa@eng.u-hyogo.ac.jp

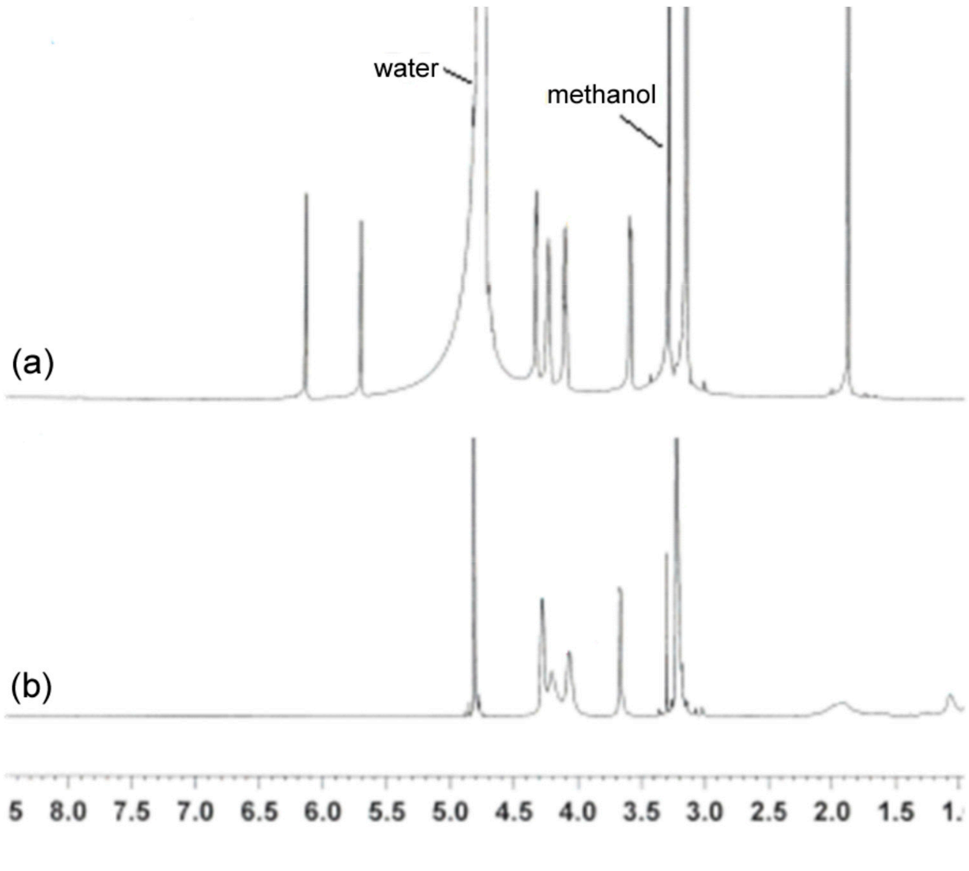

Figure S1. ^{1}H NMR spectra for MPC (**a**) before and (**b**) after polymerization in a water/methanol mixed solvent (17.0 mL, 9/1, v/v). The solution was added D_2O to lock the NMR equipment. The NMR measurements were performed at room temperature.

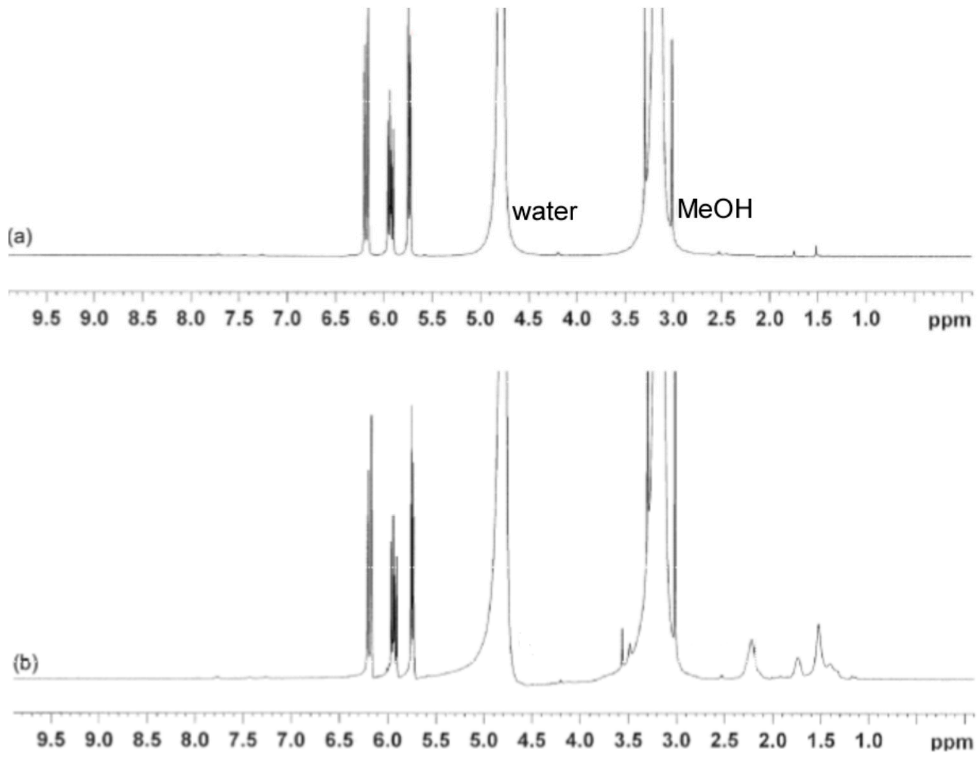

Figure S2. ^{1}H NMR spectra for acrylic acid (**a**) before and (**b**) after polymerization in methanol. The solution was added D_2O to lock the NMR equipment. The NMR measurements were performed at room temperature.

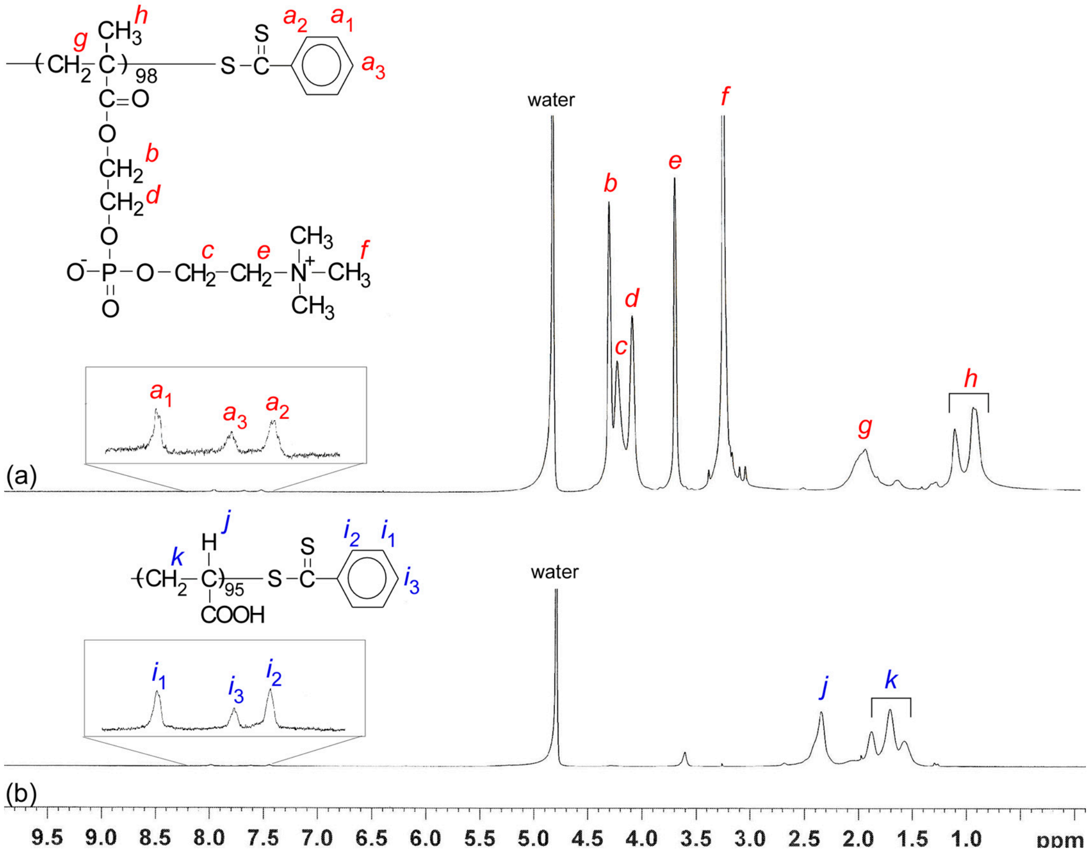

Figure S3. ^{1}H NMR spectra for (**a**) PMPC and (**b**) PAAc in D_2O at 20°C. Assignments are indicated for the resonance peaks.

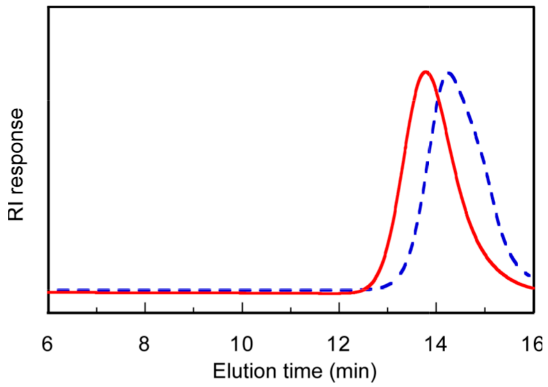

Figure S4. GPC elution curves for PMPC (—) and PAAc (---) using the mixed solvent of 50 mM phosphate buffer at pH 9 and acetonitrile (9/1, *v/v*) as an eluent at 40°C.

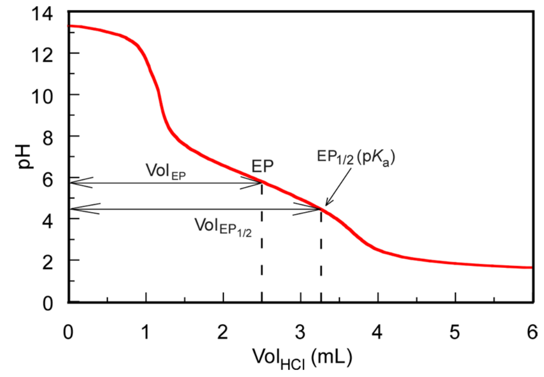

Figure S5. EP and $EP_{1/2}$ positions on the PAAc titration curve at C_p = 5.0 g/L titrated against HCl in 0.1 M aqueous solution at 25°C: Firstly, PAAc was dissolved in 0.1 M NaOH at C_p = 5.0 g/L.

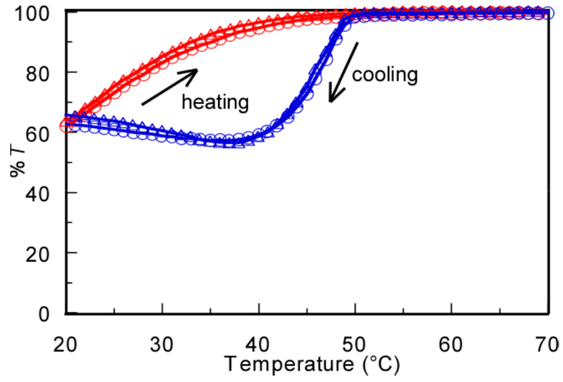

Figure S6. Percent transmittance (%*T*) at 700 nm for PMPC/PAAc with f_{AA} = 0.85 mixed aqueous solutions at pH 3, C_p = 0.5 g/L, and [NaCl] = 0.1 M as a function of temperature with the 2nd (circle) and 3rd (triangle) heating (red) and cooling processes (blue).
